# A Micro-Machined Microphone Based on a Combination of Electret and Field-Effect Transistor

**DOI:** 10.3390/s150820232

**Published:** 2015-08-18

**Authors:** Kumjae Shin, Junsik Jeon, James Edward West, Wonkyu Moon

**Affiliations:** 1 Department of Mechanical Engineering, Pohang University of Science and Technology (POSTECH), Pohang 790-784, Korea; E-Mail: forhim13@postech.ac.kr; 2 Doosan infracore, dong-gu, In-cheon 401-702, Korea; E-Mail: junsik.jeon@doosan.com; 3 Department of Electrical & Computer Engineering, Johns Hopkins University, Baltimore, MD 21218, USA; E-Mail: jimwest@jhu.edu

**Keywords:** MEMS microphone, field effect transistor, electret

## Abstract

Capacitive-type transduction is now widely used in MEMS microphones. However, its sensitivity decreases with reducing size, due to decreasing air gap capacitance. In the present study, we proposed and developed the Electret Gate of Field Effect Transistor (ElGoFET) transduction based on an electret and FET (field-effect-transistor) as a novel mechanism of MEMS microphone transduction. The ElGoFET transduction has the advantage that the sensitivity is dependent on the ratio of capacitance components in the transduction structure. Hence, ElGoFET transduction has high sensitivity even with a smaller air gap capacitance, due to a miniaturization of the transducer. A FET with a floating-gate electrode embedded on a membrane was designed and fabricated and an electret was fabricated by ion implantation with Ga^+^ ions. During the assembly process between the FET and the electret, the operating point of the FET was characterized using the static response of the FET induced by the electric field due to the trapped positive charge at the electret. Additionally, we evaluated the microphone performance of the ElGoFET by measuring the acoustic response in air using a semi-anechoic room. The results confirmed that the proposed transduction mechanism has potential for microphone applications.

## 1. Introduction

Growth in the demand for highly-sensitive microphones, coupled with advances in micromachining technology, has led to the development of many miniature acoustic pressure sensors. Micromachining and lithography techniques permit accurate control over the dimensions, yielding reproducible sensors, while offering the prospect of inexpensive, compact, mass-produced devices. Recently, micro-machined microphones, also called microelectromechanical systems (MEMS) microphones, have emerged as structures that can meet the requirements for integrated mobile device applications in the information technology (IT) hardware industry [1]. Although some MEMS microphones have already been developed, size limitations requiring microphones to be small lead to challenges in terms of frequency response and sensitivity.

The signal transduction mechanism of commercially-available MEMS microphones is typically capacitive. Capacitive microphones are usually composed of an elastic diaphragm and a perforated rigid back-plate, which constitutes a pair of sensing electrodes that behave like a variable capacitor. Deformation of the diaphragm due to variation in pressure leads to a corresponding change in the capacitance, *C_e_*, and to an induced charge at the electrodes via the application of a direct-current (DC) polarization voltage across the electrodes. In an externally-polarized condenser microphone, the polarizing voltage is applied across a very large resistance, *R_e_*. The variation in capacitance is measured from the change in the voltage across the resistor. The amplitude of this voltage signal will have a low-frequency cut-off determined from the time constant 1/*R_e_C_e_* due to the finite resistance. Therefore, further miniaturization of conventional microphones has led to a reduced size, which ultimately decreases the capacitance, *C_e_*, which increases this cut-off frequency. This frequency limit can be overcome via careful preamp design or by replacing the DC bias voltage with a high-frequency AC bias voltage [2].

Here, as an alternative to capacitive transduction, we report a transduction mechanism that is based on changes in electric field in the separation between an electret and the gate area of a field-effect transistor (FET). A FET has a large input impedance and a small output impedance. The large input impedance makes the FET very sensitive to the electric field at the gate terminal without a current, and the small output impedance facilitates straightforward signal conditioning and processing. The electret carries a permanent electric charge, which is isolated via a dielectric layer, allowing it to generate an external electric field. The structure is composed of an elastic diaphragm with the FET at the center, and an air gap and the electret placed on a rigid body. Due to this structure, we call this transducer an electret gate on FET, or ElGoFET transducer. Displacement of the diaphragm leads to a change in the separation distance between the FET and the electret, which results in a change in the electric field across the air gap and, hence, a change in the source-drain current.

The FET is integrated directly into the region where the electric field exists due to the electret, and this structure enables changes in the source-drain current without electrical loss such as parasitic effects. As a result, the FET can be used to measure the primary relative position of the diaphragm to the bound charge in the electret without signal degradation due to externally connected components such as a resistor. Therefore, this transduction mechanism will be sensitive even at low frequencies. The concept of this transduction mechanism using a FET and electret was first reported by Suzuki *et al.* [3]. Although the principle of transduction was analyzed, and the realization of the first microphone using a large foil electret diaphragm with a diameter of about 1 cm was reported, this did not demonstrate sufficient sensitivity for detecting sound. Bergveld *et al.* [4,5,6] conducted a theoretical analysis of various transduction structures using a FET and an electret for sensing applications. A simple sensitivity equation was formulated, and the sensitivities of transduction structures were compared. MEMS transducers using MEMS fabrication technology were realized. Sufficient sensitivity for audible sound pressure was again, not demonstrated; however, a differential pressure sensor suitable for high static pressures around 10 × 10^5^ Pa was evaluated.

Studies have been conducted by several groups on the application of the proposed transduction mechanism to a microphone that detects acoustic sound. Among them, Kuhnel *et al.* [7,8] showed remarkable results. The authors used a suspended gate structure in which the FET was located on a substrate and a biased electrode was deposited on a nitride membrane separated by an air gap. Strictly, a biased electrode was used instead of an electret. One difference between this transduction and transduction using a FET and electret is the source of the electric field, which arises from induced charge in the biased electrode. Although a microphone using a suspended gate structure was demonstrated experimentally, and operated over a frequency range of 0.1–20 kHz, the problem of noise still remained that comes from the change in the electric field generated using induced charge. In the transduction structure, there is no electrical shielding, and the induced charge distribution is easily disturbed by external noise. Therefore, to achieve high sensitivity relative to noise, a large bias voltage and electrical shielding are essential.

Recently, Graz *et al.* [9] reported a flexible ferroelectret FET structure that was sensitive to the electric field from a ferroelectret, where the ferroelectret was formed of a cellular dielectric with an internal bipolar electret charge. They fabricated a FET with an extended gate, which was separated from the ferroelectret by an air gap. The induced charge on the extended FET gate electrode by the ferroelectret modulates the channel of the FET. This transduction structure is interesting since it adapts a novel ferroelectret material, which has both ferroelectric and electret properties. However, a loss of sensitivity due to stray capacitance between the large extended gate electrode and the substrate cannot be avoided using this sensing structure. This transducer, as a pressure sensor, exhibited an acoustic pressure sensitivity of ~0.1 mV/Pa, with a DC bias of +8 V applied to the top of the ferroelectret via a resistor.

Prior reports of transducers using FET and electret have not yet demonstrated sound detection, but focused on pressure-sensing applications, possibly due to difficulties in realizing both the FET and electret. The objective of the present study was to realize the first microphone using a FET and electret by developing a fabrication process, particularly a binding process. A FET and electret were fabricated on physically separate substrates, and assembled on the top of each other via substrate-substrate binding. This enabled the stable fabrication of two substrates, avoiding the problem of damage to the FET due to charging during fabrication of the electret. In particular, while binding the two substrates, the electric field rising from the electret into the FET gate was observed from the drain current of the FET to characterize both the FET and the electret. This is an important aspect of evaluating the effect of the electric field into the gate of the FET, and finding the operating point of the ElGoFET microphone. After confirming a static electric field inducing the channel of the FET, the frequency response and linearity of the ElGoFET microphone was measured. Although the structure binding of the two substrates has cost implications due to the need to fabricate two wafers, this structure may introduce the possibility of a CMOS-processed application-specific integrated circuit (ASIC) to the ElGoFET microphone. The integration of the ASIC circuit may result in lower intrinsic noise.

The present paper is organized as follows: the concept and working principle of the ElGoFET microphone is presented in Section 2. Section 3 presents the fabrication of the ElGoFET microphone. The convertor circuit is described in Section 4. The bonding process for the ElGoFET microphone is described in Section 5. The evaluation of the ElGoFET microphone is presented in Section 6. Finally, the results are summarized in Section 7.

## 2. ElGoFET Microphone—Concept and Working Principle

**Figure 1:**
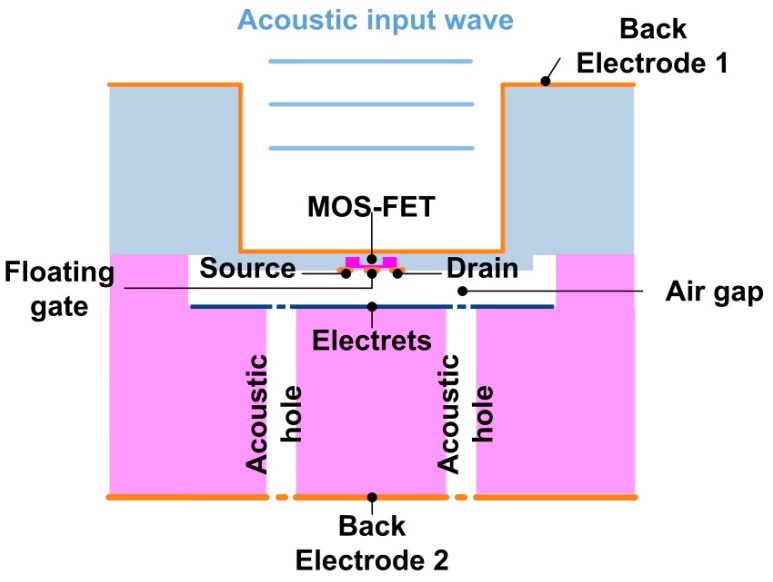
The structure of the proposed ElGo FET microphone.

The ElGoFET microphone consisted of two silicon substrates (see Figure 1). The bottom substrate contained the electret, termed the electret substrate. The top substrate contained the FET embedded on the membrane, termed the FET membrane substrate. The two substrates were aligned using mechanical alignment, so that the floating gate of the FET and electret faced each other. The substrates were mechanically clamped in the microphone housing (this process is discussed in more detail below). In this configuration, the FET is located in the electric field due to the electret.

In the FET membrane substrate, an *n*-type depletion mode MOSFET was formed on the membrane, which was 5 μm thick and 1.2 mm in diameter. The membrane material was *p*-type silicon, the source and drain regions were formed of highly-doped *n*-type regions, and the gate area was formed of a lightly-doped *n*-type region. The floating gate metal layer was deposited on the gate oxide. A body electrode was deposited at the backside of the membrane (indicated as Electrode 1 in Figure 1), which provides a reference for the electric field to the electret. In the electret substrate, the Si substrate was etched to create a 5 µm wide air gap, and the electrical charge was trapped at the 500 nm thick oxide layer. Since the electret layer was fabricated by implanting Ga^+^ ions over the entire area of the oxide layer, the electric field due to the floating gate is assumed to be uniform over the electret layer. Acoustic ventilation holes were defined on the electret substrate, allowing the compressed air in the gap to flow out and allowing the membrane to move. Electrode 2 was deposited on the backside of the electret substrate.

The transduction mechanism is based on changes in the electric field in the air gap in response to displacement of the gate of the FET relative to the electret. Deflection of the membrane due to an incident acoustic pressure wave leads to a change in the separation between the electret and the floating gate of the FET, which alters the electric field at the floating gate across the small air gap. In this manner, the floating gate potential changes in response to displacement of the membrane. The charge in the channel depends on the electric field. The induced charge at the floating gate changes in response to the floating gate potential, which leads to a change in the charge density in the channel of the FET, and hence, modulates the channel resistance. The source-drain current *I_d_* varies as a function of the electric field across air gap, *E_gap_*. Consequently, *I_d_* is modulated by the separation distance, *d_gap_*.

**Figure 2:**
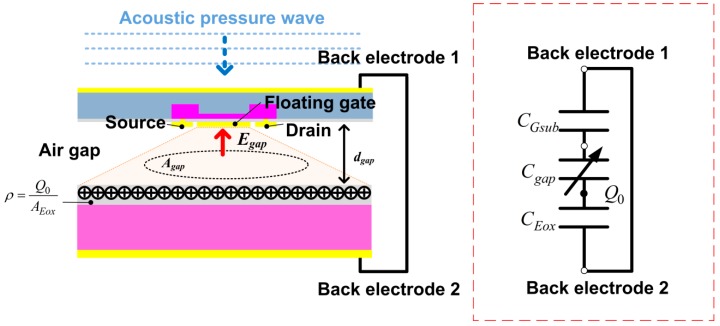
Schematic representation of the ElGo FET configuration and its electrical model.

The ElGoFET microphone can be modeled electrically as series capacitors shown by the red box in Figure 2. To create a reference potential for the electret surface, body electrodes 1 and 2 were connected: these two electrodes are referred to here as the reference electrode for the FET membrane, and the reference electrode for the electret, respectively. The capacitance between the floating gate terminal and body electrode 1 is *C_Gsub_*, the capacitance between the floating gate electrode and electret is *C_gap_*, capacitance between electret layer and body electrode 2 is *C_Eox_*, and *Q_0_* is the charge on the electret. The electric field strength at the floating gate electrode is directly related to the quantity of charge at the floating gate by *D_F.G_ = εE_F.G_*, where *D_F.G_* is the electric flux density (C/m^2^) at the floating gate and *E_F.G_* is the electric field (V/m) at the floating gate. The quantity of charge amount is related to the potential at the floating gate by *Q_F.G_ = C_Gsub_V_F.G_*. Therefore the electric field at the floating gate is related to the potential at the floating electrode.

In the static state, the potential at the floating gate is expressed as the following equation composed of capacitances.

(1) $$V_{F.G}=\frac{C_{gap}\left(Q_{0}\right)}{C_{gap}C_{Gsub}+C_{Eox}C_{Gsub}+C_{Eox}C_{gap}}$$

The existence of bound charge *Q_0_* in Equation (1) allows generation of electric field across all of the capacitor components without an external bias voltage. The surface potential ***V_s_*** of the electret layer made by ion implantation is several tens of volts [10]. This value is evaluated by using *Vs = Q_0_/C_Eox_*, and is sufficient to generate a high electric field in an air gap of a few microns. From Equation (1), the floating gate potential remains constant, assuming large gate resistance. By locating the FET itself in the region where the electric field exists due to the electret, the FET is sensitive to both consistent and varying electric fields. This shows that ElGoFET transduction is able to detect the quasi-static behavior of the membrane and very low frequency acoustic waves.

A change in the separation between the electret and floating gate, *d_gap_*, results in a change in the gap capacitance *C_gap_* because the gap capacitance can be defined as *C_gap_ = ε_gap_A_gap_/d_gap_*, where *A_gap_* is the effective area of gap capacitance and *ε_gap_* is the permittivity of air. Accordingly, the electric field *E_gap_* also changes in response to variation in *d_gap_*, as does the electric potential at the floating electrode *V_F.G_*. Therefore, changes in the potential at the floating gate in response to variations in the gap change may be expressed by differentiating Equation (1) with respect to *d_gap_*:

(2) $$\frac{dV_{F.G}}{dd_{gap}}=\frac{\left(Q_{0}C_{Eox}C_{Gsub}\right)}{{\left(C_{gap}C_{Gsub}+C_{Eox}C_{Gsub}+C_{Eox}C_{gap}\right)}^{2}}\frac{-C_{gap}}{d_{gap}}$$

The equation of *dV_F.G_/d_dgap_* is also composed of capacitance relations. If the equation is rewritten the form of Equation (3),

(3) $$\frac{dV_{F.G}}{dd_{gap}}=\frac{-Q_{0}}{\varepsilon_{gap}A_{gap}}\frac{\frac{C_{gap}}{C_{Eox}}\frac{C_{gap}}{C_{Gsub}}}{{\left(\frac{C_{gap}}{C_{Eox}}+1+\frac{C_{gap}}{C_{Gsub}}\right)}^{2}}$$

It is noted that *dV_F.G_/d_dgap_* is modeled as a function of the ratio of capacitances: *C_gap_*/*C_Eox_* and *C_gap_/C_Gsub_*. This recast equation shows that the ratio of capacitances has more influence on *dV_F.G_*/*d_dgap_* than the gap capacitance itself, unlike general capacitive transduction. Even if the gap capacitance, *C_gap_*, is small, if the capacitance ratios, *C_gap_*/*C_Eox_* and *C_gap_*/*C_Gsub_*, are sufficiently large, the sensitivity can be modified and increased. Therefore, considering that gap capacitance depends on the size of the transducer, ElGoFET transduction has high sensitivity even at reduced size by miniaturization.

The floating potential obtained enhances the FET channel and the source-drain voltage makes the channel current flow. The FET embedded on the membrane operates in a similar manner to a MOSFET due to the existence of the floating electrode [5]. The operating region of the FET is determined as a function of *V_DS_* and *V_F.G_*. The ElGoFET was set to operate in the saturation region, the FET had the largest trans-conductance (*i.e.*, *dI_d_*/*dV_F.G_*, was at a maximum). 

Following the MOSFET theory, the change in drain current in response to gap variation, *dI_d_*/*dd_gap_*, is defined by multiplying *dV_F.G_*/*dd_dgap_* and *dI_d_*/*dV_F.G_* using chain rule. Therefore *dI_d_*/*dd_gap_* can be expressed as the following equation.

(4) $$\frac{dI_{D}}{dd_{gap}}=\frac{-Q_{0}}{\varepsilon_{gap}A_{gap}}\frac{\frac{C_{gap}}{C_{Eox}}\frac{C_{gap}}{C_{Gsub}}}{{\left(\frac{C_{gap}}{C_{Eox}}+1+\frac{C_{gap}}{C_{Gsub}}\right)}^{2}}\cdot \mu \frac{W}{L}{C_{Gox}}^{\prime }\left(V_{F.G}-V_{T}\right)$$

where ${C_{Gox}}^{\prime }$ is the gate oxide capacitance per unit area, *V_T_* is the threshold voltage, and µ is the electron mobility. From Equation (4), it is noted that drain current variation, *dI_d_*/*dd_gap_*, follows characteristics of *dV_F.G_*/*dd_dgap_*. Therefore, the ElGoFET transduction mechanism is advantageous, because it does not require an external bias, has low frequency sensitivity and, in principle, a weak size effect. This is made possible by direct detection of electric fields due to electret using an integrated FET.

## 3. Fabrication of the ElGoFET Microphone

Fabrication consists of two main steps. In the first step, the FET membrane and electret structure are fabricated on separate wafers and released. The electret is fabricated using Ga^+^ implantation. The second step is bonding these two substrates. It is not possible to use a general wafer bonding process to construct an ElGoFET microphone as the electric field effect rising from the electret to the floating gate cannot be evaluated, because the wafer bonding process cannot be conducted with the FET in an electrically on state. In this case, FET characterization is only possible after completion of the wafer bonding process. In the present study, a substrate-substrate binding process was conducted. Because the FET is in an electrically on state during the whole binding process, the effects of the electric field from the electret on the floating gate of the FET can be evaluated by measuring the drain current change, and the FET’s operating point can be characterized.

**Figure 3:**
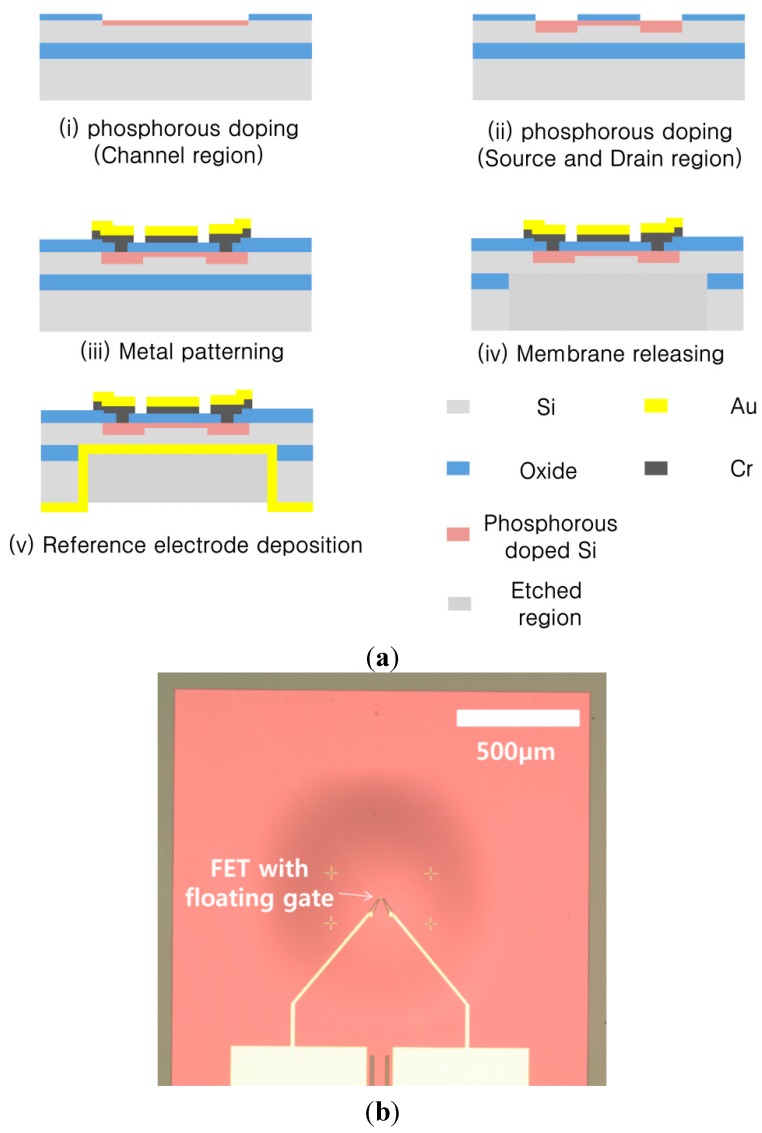
(**a**) Fabrication of an FET-membrane substrate; (**b**) Top view of the fabricated FET membrane substrate.

Here, the first step is described. The FET membrane substrate was fabricated using standard CMOS process technology, as shown in Figure 3a. We used a silicon-on-insulator (SOI) wafer, with a 5 μm thick device layer, a 1 μm thick buried oxide, and a 400 μm thick handle layer. To create the depletion region of the depletion-mode metal oxide semiconductor (MOS), the channel area was doped with phosphorous via ion implantation at 20 keV with a dose of 2 × 10^12^ cm^−2^. The source and drain region were doped at 80 keV with a dose of 1 × 10^15^ cm^−2^. The structure was then annealed at 1000 °C for 20 s. The gate dielectric was formed by thermal dry oxidation to obtain a 30 nm thick oxide layer. Contact via holes for the source and drain region were defined using optical lithography, and etched using a wet chemical etching process with buffered oxide etchant (BOE). A Cr (15 nm)/Au (150 nm) metal stack was then deposited via sputtering, where the Cr layer was used for adhesion of the Au. The electrodes were patterned using wet chemical etching. Following the fabrication of the FET, dry etching was performed on the front side to define the membrane region to form a 5 µm deep alignment key for bonding the FET membrane substrate to the electret substrate. To form the membrane structure, the backside was dry etched using deep reactive ion etching (DRIE), and the reference electrode for the FET was deposited via sputtering. The fabricated FET embedded with the membrane is shown in Figure 3b.

**Figure 4:**
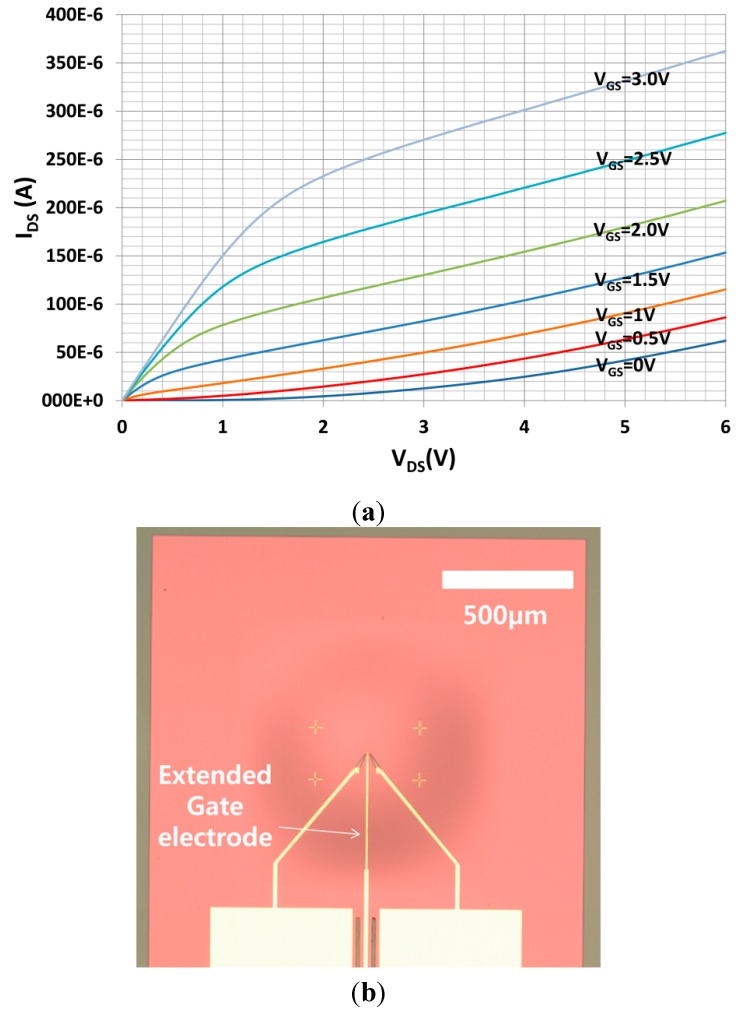
(**a**) I-V characteristics of fabricated FET membrane with a gate electrode pad; (**b**) Top view of the fabricated FET membrane with a gate electrode pad.

**Figure 5:**
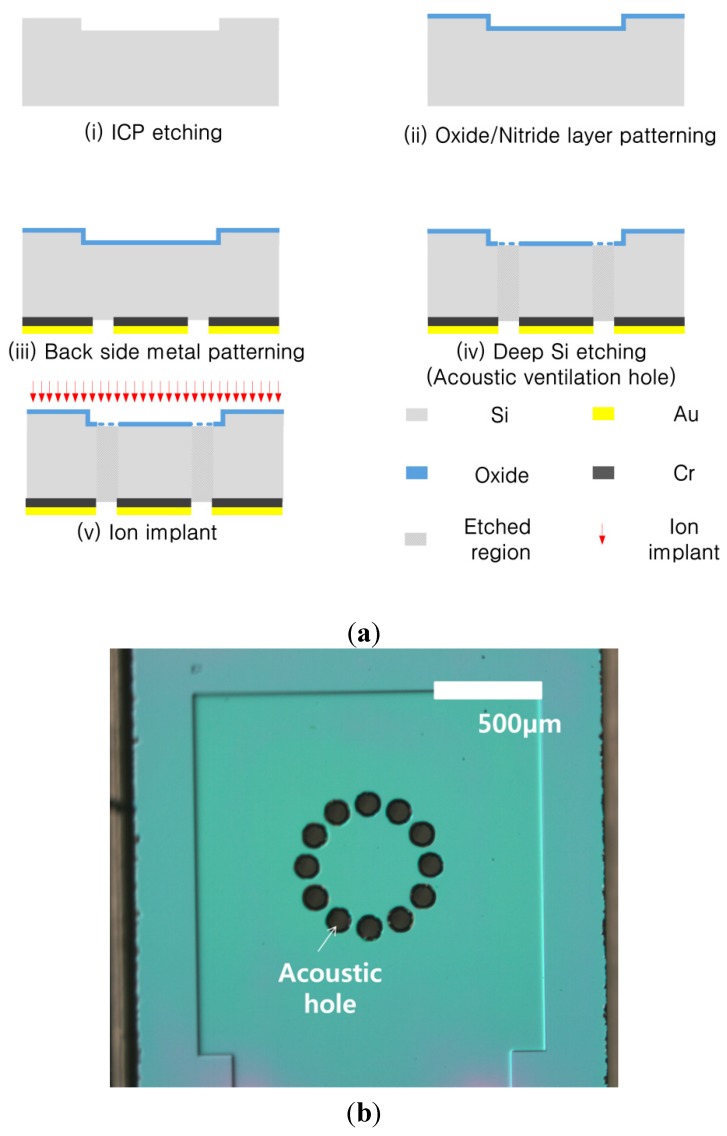
(**a**) Fabrication of an electret substrate; (**b**) Top view of the fabricated electret substrate.

The FET was characterized by measuring the source-drain current with a voltage sweep in the range of 0–6 V with various fixed gate biases using a semiconductor analyzer (HP 4156B). The resulting current-voltage (*I-V*) characteristics are shown in Figure 4a. To make these measurements, an additional gate electrode was required to apply the gate bias. The device structure used here is shown in Figure 4b; it was fabricated on the same wafer as the FET with the floating gate electrode, as shown in Figure 3b. To fabricate the electret substrate, a *p*-type Si wafer with a resistivity of 10 Ω·cm was used. As shown in Figure 5a, features on the front side of the bulk Si were etched to a depth of 10 μm using inductively-coupled plasma (ICP) etching. These determine the spacing between the membrane and electret, and the resulting air gap was 5 μm. Wet chemical oxidation was performed to create a 500 nm-thick oxide dielectric layer. The back electrode was deposited on the rear side. The bulk Si was perforated using a deep Si-etch process to make 110 μm diameter air acoustic holes [11]. The electret substrate was then released, which measured 2.4 mm × 2.4 mm, as shown in Figure 5b.

The ion-beam method was used to trap a positive electric charge in the oxide layer. Bombarding an insulator with Ga^+^ causes the specimen to accumulate excess positive charge and the bound charge layer is generated at oxide surface [10]. This was achieved using a FIB, which can achieve localized implantation. Prior to ion implantation, the sample was desiccated for 2 h at 260 °C, and then treated with hexamethyldisilazane (HMDS) vapor at 140–170 °C. Desiccation was necessary because the wafers were stored following fabrication, during which time water molecules in the air may become adsorbed onto the oxide surface, and can migrate into structural defects. The HMDS treatment provides long-term hydrophobic protection. The Ga^+^ ions were implanted at an energy of 5 keV and a dose of 2 × 10^12^ cm^−2^. The dielectric layer was exposed to the ion beam for 1 s. The electric potential and surface charge density of the fabricated electret were evaluated by assembling the FET membrane substrate and electret substrate.

## 4. Electric Peripherals (I-V Converter)

**Figure 6:**
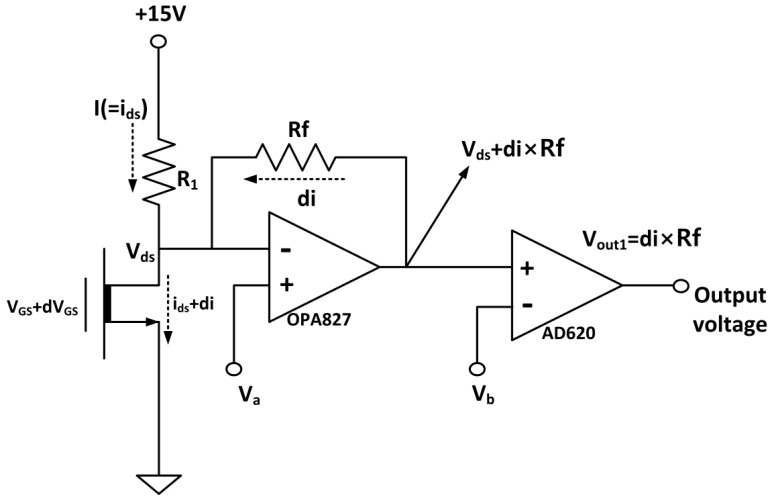
Circuit diagram of the I-V converter.

Variation in the drain current from the FET membrane was in the order of a few µA. The output signal was converted into a voltage signal using an *I-V* converter circuit to measure the output current signal from FET. Figure 6 shows a circuit diagram of the *I-V* converter. The source-drain voltage of the FET *V_DS_* was determined from *V_a_*, because the drain terminal was connected to the inverting input of an operational amplifier (op-amp). The FET therefore operates under a drain voltage for a given *V_a_*. The circuit operates with variation in the electric field of the gate oxide resulting in a signal current d*I*, which flows through the feedback resistor *R_f_*. Accordingly the *I-V* converter circuit converts the drain current of the FET to a voltage signal via the use of the feedback resistor. Since the first stage of the op-amp dominates the noise characteristics and the frequency response, we used a Texas Instruments OPA827 op-amp, which has a large frequency bandwidth that includes audible frequencies. The second stage op-amp was an AD620, and was used to eliminate the bias voltage *V_b_*, resulting in an AC output signal. The *I-V* converter was fabricated using a printed circuit board (PCB).

## 5. Microphone Assembly—Constructing ElGoFET Microphone and Discussion

In the present study, the assembly of two substrates with an electret and a FET, respectively, is adopted instead of a wafer bonding process with many electrets and FETs. It may be more difficult and time-consuming to precisely assemble the two small pieces of substrate, each of which contains an electret or a FET, but the assembly makes it possible to measure the source to drain electrical current in the FET while they are being assembled. Therefore, we can ensure, experimentally, that the proposed transduction works as expected, which is useful for the feasibility study of the device. The data measured during assembly would be used for mass fabrication of the target device using wafer bonding in the future. Appropriate drain and source voltages were applied to the FET to create the on state to evaluate the electric field effect by the electret on the floating gate.

**Figure 7:**
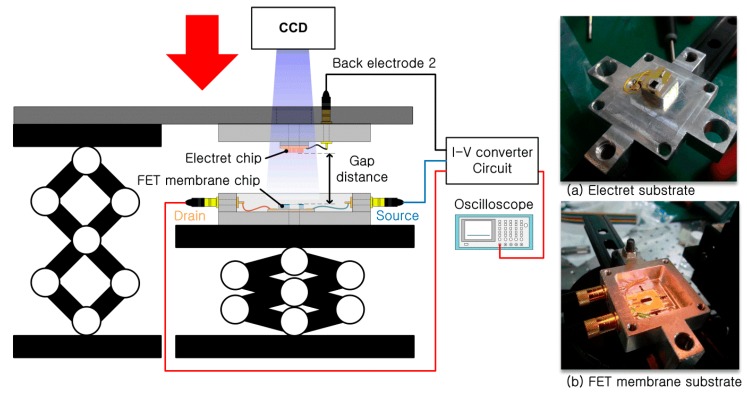
Experimental setup for mechanical bonding.

The two fabricated substrates were aligned and assembled using a mechanical manipulator. Figure 7 shows the mechanical binding experiment setup. The fabricated electret substrate and FET membrane substrate were attached to each PCB, which contained a hole that was larger than the circular membrane of the FET membrane substrate. The membrane was centered on the PCB hole, and the FET membrane was attached to the PCB using silver paste to form an electrical contact between the reference electrode and the electrode pad on the PCB, as the reference electrode was located on the back of the FET membrane. The source and drain terminals of the FET membrane were connected to the electrode pads by wire bonding. A reference electrode for the electret substrate was also located on its back. The silver paste was also used as an adhesive to connect the reference electrode of the electret substrate to the electrode pad on the PCB. The PCB for the electret substrate also had a hole in it, over which the electret was centered to obtain an optical image of the FET surface through the perforated hole of the electret substrate during the mechanical bonding process described below.

**Figure 8:**
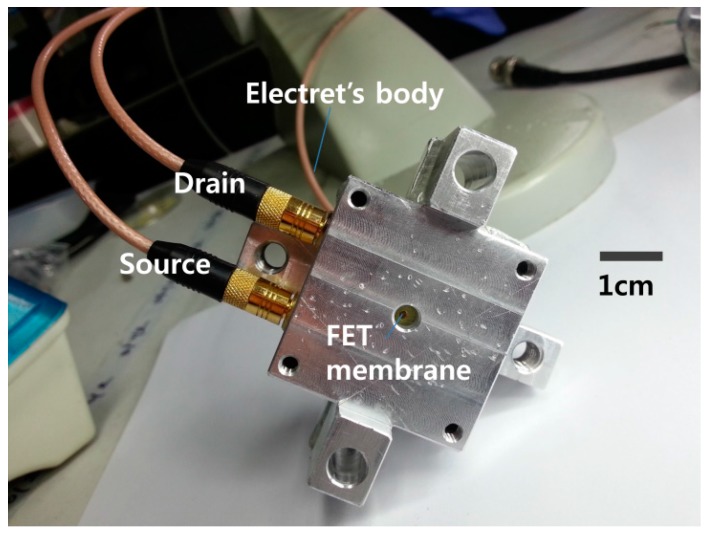
The assembled ElGoFET microphone.

**Figure 9:**
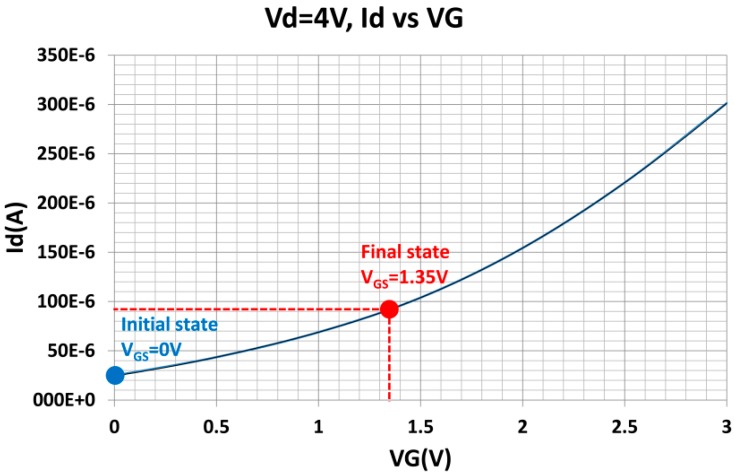
The I_DS_-V_G_ characteristic curve at V_D_ = 4 V.

The PCBs were mounted on a mechanical housing, as shown in the insets (a) and (b) of Figure 7. The electret substrate and the FET membrane substrate were mechanically bonded by assembling the two mechanical housings using a mechanical manipulator. Initially, the electret and FET membrane substrates were separated by 10 cm with the FET membrane working at *V_DS_* = 4 V and with a feedback resistance of *R_f_* = 68 kΩ. In this state, the output signal from the *I-V* converter was set as the initial voltage. The electret substrate was then moved toward the FET membrane substrate using the mechanical manipulator. When the electret substrate approached the FET membrane, the two substrates were aligned while monitoring the structure using a charge-coupled device (CCD) camera. The two substrates came into contact via their housings, and were fastened using a bolt. Figure 8 shows the assembled ElGoFET microphone. The output signal was observed using an oscilloscope as the two mechanical housings were assembled. Following assembly, the output signal of the *I-V* converter increased by 4.57 V from the initial value for the separated structures, and remained constant. The drain current change was calculated as 67.2 μA with a feedback resistance of *R_f_* = 68 kΩ. From this experiment, it is possible to evaluate the effect of the electric field rising from the electret to the floating gate of the FET. After complete assembly, ***I_DS_*** only depends on the electric field from the electret since the FET is electrically isolated from environmental conditions. Therefore the change in drain current makes it possible to evaluate the potential at the floating gate. The induced floating gate potential was estimated based on the change in the output signal. The change in the drain current as a function of the gate potential, *I_DS_*(*V_G_*) at V_D_ = 4 V, was obtained based on measured I-V characteristics of the FET shown in Figure 4a, and plotted in Figure 9. These data were fitted to a polynomial:

(5) $$I_{d}=8\times 10^{-1}{V_{G}}^{6}-8{V_{G}}^{5}+30{V_{G}}^{4}-40{V_{G}}^{3}+40{V_{G}}^{2}+30V_{G}+20\;\left(\mu A\right)$$

The potential at the floating gate of the FET was calculated assuming that the initial gate potential was zero. Therefore the operating point of the ElGoFET was determined to be *V_FG_* = 1.35 V (see Figure 9) with *V_D_* = 4 V.

From this experiment, the drain current changes and is later constant, suggesting that the ElGoFET transduction mechanism does not require an external bias voltage. Furthermore, the ElGoFET transduction is sensitive to very low frequencies (*i.e.*, a DC input signal). So the low-frequency cut-off that is typical of capacitive microphones is not an issue for the ElGoFET device.

## 6. Acoustic Experiment Result and Discussion

**Figure 10:**
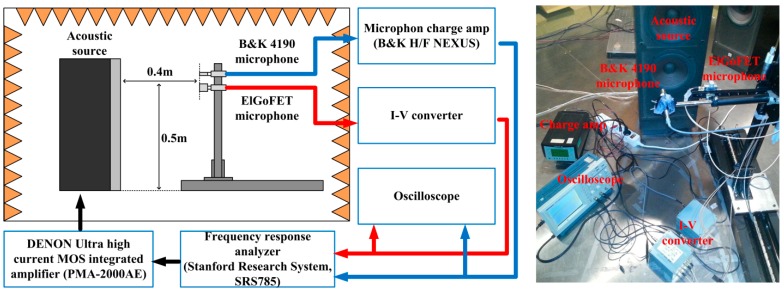
The acoustic measurement setup.

The acoustic measurement setup used to measure the receiver characteristics of the fabricated ElGoFET microphone was installed in a semi-anechoic room, as shown in Figure 10. The acoustic sources were a JBL G500 loudspeaker and an SR-100A25 mid-range loudspeaker, which were used in the ranges of 50–2000 Hz and 2–20 kHz, respectively. The ElGoFET microphone and a B&K 4190 reference microphone were installed at similar measurement locations, both 0.4 m from the loudspeaker and 0.5 m from the floor of the semi-anechoic room. The acoustic signal was measured using the reference microphone, and the signal from the ElGoFET microphone was calibrated using the signal from the reference microphone. The signal generated at the output port of the dynamic fast Fourier transform (FFT) signal analyzer (SRS785) was amplified using a Denon power amplifier and transmitted to the loudspeaker. The reference microphone was set with sensitivity of 1 V/Pa, and the ElGoFET microphone was converted into a voltage signal using the *I-V* converter with a feedback resistance of *R_f_* = 680 kΩ. Here, the feedback resistance was ten times larger than used during the assembly experiment, to observe a clear output signal. The signals of the reference microphone and the ElGoFET microphone were monitored using an oscilloscope and analyzed simultaneously using the frequency response analyzer.

**Figure 11:**
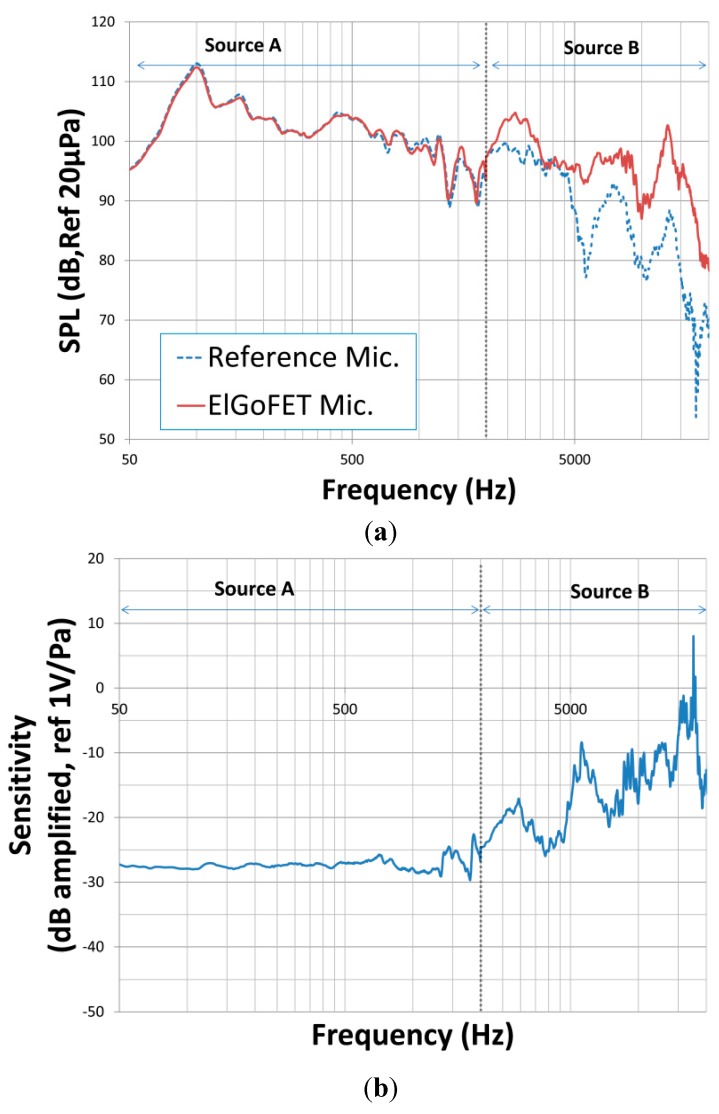
(**a**) A comparison of the output signal of the reference microphones and the ElGoFET microphone with acoustic signals generated from the loudspeaker; and (**b**) the frequency response of the ElGoFET microphone compared with that of the reference microphone in dB units relative to 1V/Pa.

With frequencies swept across the range 50 Hz to 20 kHz, the sound pressure level measured using the reference microphone and the ElGoFET microphone is shown in Figure 11a. Figure 11b shows the measured sensitivity of the ElGoFET microphone. In the frequency range of 50 Hz to 2 kHz, the response of the two microphones was similar; however, in the range 2–20 kHz, the ElGoFET microphone was more sensitive than the reference microphone.

Particularly at frequencies greater than 1 kHz, the peaks and nulls in the frequency response to the ElGoFET microphone are attributed to vibrational modes of the mounting structure. The source of these spikes is unclear; however, similar features have been reported previously [12]. Complex interference from reflected acoustic signals, including those reflected from the floor of the semi-anechoic room, may give rise to these patterns. Furthermore, the dimensions of the housing can lead to such artifacts. The housing of the ElGoFET microphone was larger than that of the reference microphone (the assembled ElGoFET device was cubic, and the length of each side was 3.5 cm, whereas the shape of reference microphone was cylindrical with a diameter of 1.27 cm), which results in diffraction effects in the ElGoFET microphone at lower frequencies than in the reference microphone. When a microphone is placed in a sound field, diffraction effects alter the acoustic pressure at the diaphragm if the frequency is sufficiently high that the wavelength of the signal is similar to the dimensions of the microphone [13]. Accordingly, artifacts of the housing appear in the output signal of the device when the ratio of the dimensions of the microphone to the wavelength, *d*/λ, increases. With the reference microphone, the ratio of the diameter to the wavelength at 20 kHz was *d*/λ = 0.74; however with the ElGoFET microphone, we find *d*/λ = 2.04 at 20 kHz. Therefore, we may expect diffraction effects to be more significant with the ElGoFET microphone. It appears that these complex patterns of the frequency response shown in Figure 11b are not a problem with the microphone transduction itself, but rather an issue with the packaging of the device and the experimental setup.

**Figure 12:**
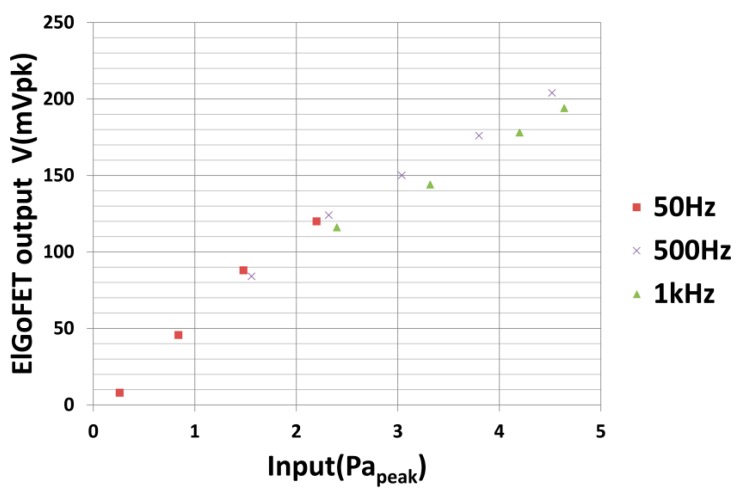
Linearity of the ElGoFET microphone.

To evaluate the linearity of the microphone, the relationship between the output signals measured using the two microphones was evaluated at several frequencies. Figure 12 shows the relationship between the amplitudes of the output signals from the reference microphone and from the ElGoFET microphone. A number of single-tone signals were used with fixed frequencies of 50 Hz, 500 Hz, and 1 kHz, and the amplitude was varied. The harmonic distortion was measured, and the peak magnitude is shown in Figure 12. The response was linear from an SPL of 97 dB at 50 Hz to an SPL of 107 dB at 1 kHz.

The fabricated ElGoFET microphone detects sound pressure waves over a frequency region from 50 Hz to 20 kHz. Although characterization of the ElGoFET microphone below 50 Hz was not conducted due to the size of the semi-anechoic room, the measured results show the potential of the ElGoFET transduction as a microphone. Modification of the experimental environment is required for accurate measurement in the low frequency region below 50 Hz. However, the potential to measure low frequency sound has been shown in the assembly experiment by evaluating the static response.

## 7. Summary and Conclusions

Capacitive MEMS microphones, now widely used, have a miniaturization limit due to the loss of sensitivity at low frequencies. This work presents a microphone (ElGoFET) using the transduction mechanism based on an electret and a FET, which can overcome the limit of miniaturization for low-frequency applications. The sensitivity of the ElGoFET is not dependent on gap capacitance alone—a quantity usually determined by the physical dimensions of the capacitive sensor—but instead depends upon the ratio of capacitance components within the ElGoFET transduction structure. Therefore, ElGoFET transduction can be used effectively to design a miniaturized microphone without the voltage biasing that is necessary in conventional capacitive sensors.

To investigate the feasibility of the proposed ElGoFET transduction mechanism for microphones, a device using this mechanism was designed, fabricated, and tested. The effects of the electric field from the electret on the FET gate were demonstrated via the assembly process of the device; a static response of the FET to the electric field from the positive trapped charge of the electret was observed. This finding demonstrates that the ElGoFET can detect the direct behavior of the acoustic diaphragm, which has the potential for wideband frequency response across regions including very low frequencies, and possibly even a quasi-static response.

The measured frequency response of the ElGoFET microphone was quite flat—within 3 dB deviation from 50 Hz to 1 kHz—with a high level of sensitivity, about 10 dB greater than normal electret microphones. Despite several peaks and nulls in the response above 1 kHz, the measured results demonstrate sufficient performance as a microphone, because these deviations can be largely attributed to several acoustic effects, such as acoustic diffraction by the large cubic housing and other experimental factors regarding the calibration setup, rather than the characteristics of the ElGoFET transduction mechanism.

This work demonstrates the potential of ElGoFET transduction for miniaturized low-cost high-performance microphones via batch processing. The ElGoFET characterization results may be used to improve the performance of the miniaturized ElGoFET microphone, which will be fabricated using a batch-process with wafer-bonding in future work. Precise characterization of the response at very low frequencies (below 50 Hz) will also be conducted with the newly fabricated microphone. These developments are expected to lead to high sensitivity at low frequencies at a miniaturized scale, which cannot be achieved in conventional capacitive MEMS microphones.
